# Ocular and Neurological Sequelae in Long COVID: Dry Eye, Asthenopia, Sleep Disorders, Asthenia, and Restless Legs Syndrome—A Case Report with Literature Review

**DOI:** 10.3390/life15081289

**Published:** 2025-08-14

**Authors:** Mario Troisi, Salvatore Troisi, Livio Vitiello, Diego Strianese, Carlo Bellucci, Michele Rinaldi, Luca D’Andrea, Ciro Costagliola

**Affiliations:** 1 Eye Clinic, Department of Neurosciences, Reproductive and Odontostomatological Sciences, University of Naples Federico II, 80131 Naples, Italy; 2 Ophthalmologic Unit, Salerno Hospital University, 84100 Salerno, Italy; 3 Eye Unit, “Luigi Curto” Hospital, Azienda Sanitaria Locale Salerno, 84035 Polla, Italy; 4 Ophthalmology Unit, Department of Medicine and Surgery, University Hospital of Parma, 43126 Parma, Italy

**Keywords:** asthenia, asthenopia, dry eye, long-COVID, restless legs syndrome, sleep disorders

## Abstract

This case report presents a unique constellation of symptoms—including dry eye disease, visual and general asthenia, sleep disturbances, and restless legs syndrome—in a patient with a recent history of coronavirus disease 2019 (COVID-19) infection. While these symptoms have individually been associated with either COVID-19 or long COVID, their concurrent presentation and the simultaneous, positive response across all manifestations to a combined therapeutic regimen have not been previously described in a single case. The patient demonstrated notable improvement in both ocular and systemic symptoms following a six-week treatment with topical tear substitutes and oral administration of melatonin, and a multivitamin supplement including B-complex vitamins, antioxidants, and neuroprotective agents (Colinplus Delta^®^, Farmaplus Italia Srl, Via Giovanni Porzio 4, 80143 Napoli, Italy). This response suggests a possible shared pathophysiological mechanism underlying these manifestations, potentially involving post-viral neuroinflammation, immune dysregulation, oxidative stress, or autonomic dysfunction. This case report highlights the need for an increased awareness of the interconnected nature of ocular and neurological symptoms in long COVID and supports further research into non-invasive, multimodal treatment strategies for this emerging clinical spectrum.

## 1. Introduction

Coronavirus disease 2019 (COVID-19) is a pandemic disease caused by the severe acute respiratory syndrome coronavirus 2 (SARS-CoV-2). Patients generally show flu-like symptoms, with high fever and breathing problems. The disease was classified as a mild, severe, or critical illness based on its clinical presentation. Most patients (>80%) had mild illness (from mild pneumonia to no symptoms), while 14% suffered from serious forms which manifested with dyspnea, an increased respiratory rate, a decrease in blood oxygen saturation (SpO2 < 93%), and pulmonary infiltrates [1]. A minority of patients (5%) developed a severe, life-threatening form, with the development of respiratory failure, septic shock and/or multiorgan failure [1] and ground-glass-like, opaque pulmonary formations on chest computed tomography scans [2]. In the last three years, variants of the virus that were generally less severe and more diffusible have developed; in particular, recent studies revealed that Omicron is less severe than other variants, with a risk of hospitalization ranging from 15% to 80% lower than the Delta variant [3].

While diagnostic and therapeutic efforts have mainly focused on the respiratory and hematological complications of the disease, several ocular implications have also emerged over time [4]. Some cases of COVID-19 disease showed ocular surface alterations with possible viral detection in tear fluid [4,5,6]. The most common ocular findings are bilateral conjunctival hyperemia, epiphora, and foreign body sensation, followed by itching, eyelid swelling, and mucopurulent discharge [7]. The exact incidence of conjunctivitis in COVID-19 patients is still unclear (range 0.8–31.6%) [8,9]. Numerous theories have been proposed to clarify how coronavirus can end up in ocular fluids: (1) conjunctiva as the site of direct inoculation by infected droplets, (2) the nasolacrimal duct as a migration route of the virus to the upper respiratory tract, or (3) hematogenic infection of the tear glands [10]. However, different studies confirm that in patients affected by ocular surface inflammations, there is a low percentage of positive conjunctival swab results [10]. In a recent review, viral RNA was detected in conjunctival secretions and tears in three cases out of 120 (2.5%) COVID-19 patients, with a range of 0–8% [11]. Another study reported a high percentage of viral detection in the tear fluid of COVID-19 pneumonia patients admitted to sub-intensive units, probably for conjunctival contamination by respiratory fluids due to face mask or helmet use [5]. Probably, in negative cases, the viral load was below the threshold of test detection, while some patients had already started systemic antiviral therapy before the swab [9]. Moreover, the eye may serve as a site of virus replication and as a gateway for virus transfer to extraocular sites, particularly to the respiratory system [12]. Ocular symptoms sometimes persist during the following months and are considered a possible complication of the infection, called long COVID (LC) [13].

Gambino et al. performed an evaluation of the ocular surfaces of post-COVID-19 patients compared to a control group of healthy subjects, and a statistically significant increase in dry eye disease emerged in both subjective and objective assessments [14]. Furthermore, patients with higher viral loads have greater risks of ocular surface disorders; in fact, meibomian gland disease and ocular surface staining are more common and severe in post-COVID-19 patients, while patients requiring supplementary oxygen are more likely to show tear film instability [15].

The prevalence of sleep disturbance was 33.3–84.7% in hospitalized COVID-19 patients. Factors linked to sleep disorders are as follows: neuronal injuries directly and indirectly caused by SARS-CoV-2 infection; physical discomforts including cough, fever, pain, and dyspnea; perception of the disease severity; social isolation; and adverse effects of medication, such as corticosteroids, beta-blockers, and nonsteroidal anti-inflammatory drugs. In addition, environmental factors including noise, abnormal light exposure, patient care activities, diagnostic and treatment procedures, and circadian rhythmicity alterations are related to sleep disorders [16]. Fatigue and sleep disturbances have also been reported as long-term manifestations after acute infection, but no correlation with ocular symptoms has been reported so far [17].

In this case report, the authors describe the management of a patient with a history of acute SARS-CoV-2 infection who subsequently developed persistent dry eye and asthenopia, accompanied by systemic symptoms including asthenia, sleep disturbances, and restless legs syndrome (RLS); in addition, the results of the adopted therapeutic approach on the ocular and systemic symptoms, also in relation to data in the literature, are analyzed.

## 2. Case Description

**Figure 1:**
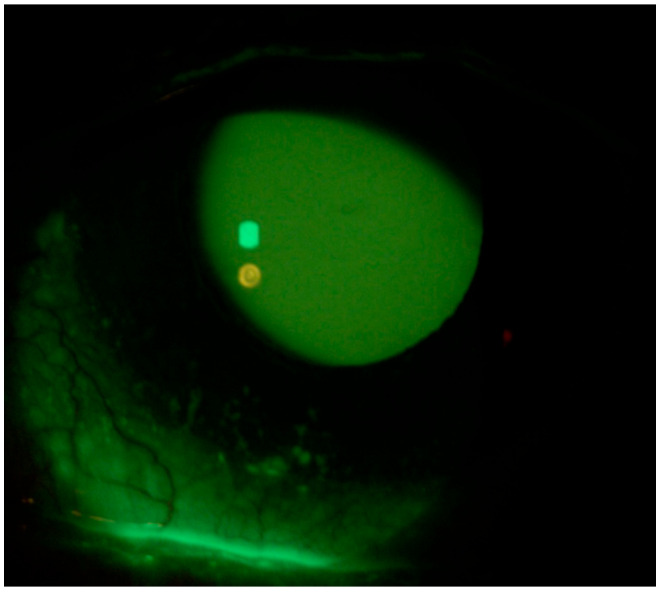
Right eye at presentation (Fluotest).

**Figure 2:**
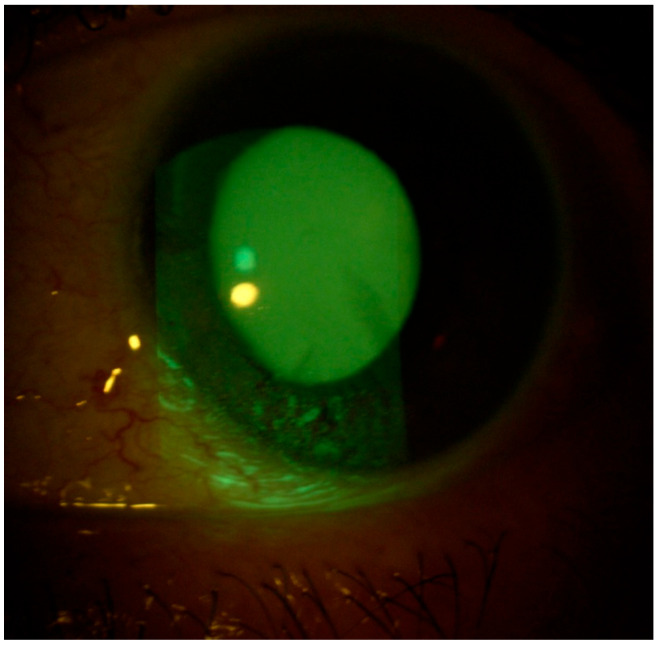
Left eye at presentation (Fluotest).

A 59-year-old Caucasian male patient with a past medical history of mild dyslipidemia received three doses of the Cominarty (Pfizer-BioNTech) vaccine for SARS-CoV-2 (with the last dose administered 7 months before coming into our observation). The patient was evaluated for the presence of about six weeks’ ocular burning in both eyes, which was associated with foreign body sensation, and symptoms of asthenopia—characterized by visual fatigue, ocular discomfort, blurred vision, and frontal headaches triggered or worsened by prolonged near tasks, particularly during video terminal work (VDT). These symptoms began a few days after the patient contracted COVID-19. He had a fever in his first three days of the COVID-19 illness, which was associated with persistent upper respiratory congestion; molecular buffer negativization was detected 9 days after the first positive RT-PCR test. He also reported that he had never noticed any previous ocular surface disturbances or foreign body sensations. Ophthalmological examination showed a best-corrected visual acuity of 0.0 LogMar in both eyes; reduced lacrimal meniscus in both eyes; punctate epitheliopathy in the lower corneal sectors of right (CLEK: 2) (Figure 1) and left eye (CLEK: 3) (Figure 2), according to the Collaborative Longitudinal Evaluation of Keratoconus (CLEK) grading system; slight cortical clouding of the lens in both eyes; normal ocular ductions; mild exophoria and convergence deficit, with a proximal point of convergence (PPC) of 38 cm; and no pathological findings in the fundus oculi in both eyes.

A tear breakup time (TBUT) test had a result of 3 s in the right eye and of 2 s in the left eye; on the other hand, a Schirmer I test had a result of 8 mm in the right eye and of 6 mm in the left eye, with an Ocular Surface Disease Index (OSDI) of 36.

**Figure 3:**
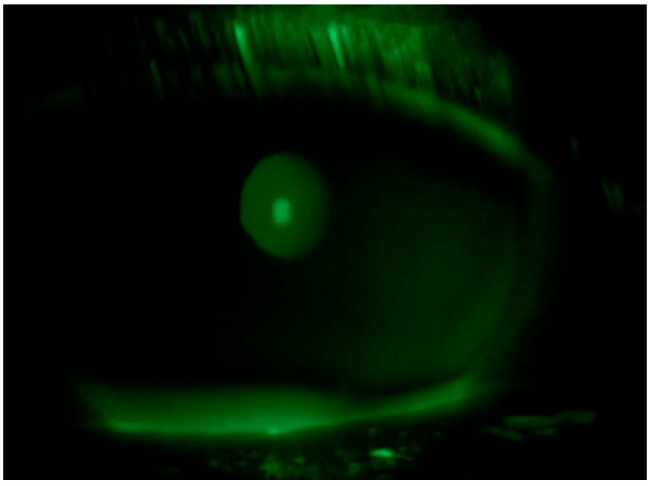
Fluotest on 10th day in the right eye.

**Figure 4:**
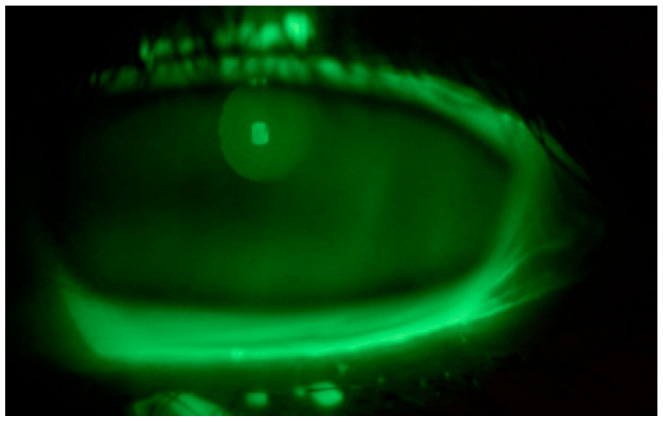
Fluotest on 10th day in the left eye.

Tear substitute composed of hyaluronic acid, vitamin B2, vitamin E-TPGS, methylsulfonylmethane, and amino acids four times a day in both eyes and an antibiotic ointment (tetracycline + sulfamethylthiazole) before bedtime were prescribed; an ophthalmological follow up was performed after ten days: negative corneal Fluotest in both eyes (Figure 3 and Figure 4); TBUT right eye: 6 s, TBUT left eye: 5 s; Schirmer I test right eye: 10 mm, Schirmer I test left eye: 9 mm; and OSDI: 25.

The patient reported an improvement in dry eye symptoms but persistent difficulties in activities involving a personal computer (PC), reading and smartphone use, with easy tiredness, frontal headache, fleeting loss of vision, and occasional diplopia. He also reported that, since he contracted the infection, he suffered from insomnia and nighttime RLS and, more generally, the condition of asthenia. The patient’s reading speed, assessed using the International Reading Speed Texts (IReST), was significantly reduced at 95 words per minute (wpm), consistent with reported difficulty in sustained near-vision tasks.

The patient was started on an oral therapy with a multivitamin product—Colinplus Delta^®^ (Farmaplus Italia Srl, Via Giovanni Porzio, 4, 80143 Napoli, Italy)—including Vitamin B1, B5, B6, B12, C, D3, and E, Anthocyanosides, Choline, Reishi (a medicinal mushroom extract), Coenzyme Q10, and SOD, combined with zinc gluconate (17.4 mg) and selenium methionine (100 mcg) in the evening. He also continued the instillation of a tear substitute containing hyaluronic acid, vitamin B2, vitamin E-TPGS, methylsulfonylmethane, and aminoacids four times a day in both eyes. Additionally, melatonin (2 mg) was also administered orally.

After 6 weeks of treatment, the patient reported a significant improvement in ocular symptoms and the resolution of the irritative disorders and asthenopia, with a complete resumption of work and daily activities. Ophthalmologic examination at 6 weeks showed a negative corneal Fluorescein test in both eyes; TBUT right eye: 9 s, left eye 8 s; Schirmer I test: right eye: 10 mm, left eye: 9 mm; and OSDI: 11. The patient also reported a subjective improvement in visual comfort and reduced effort during reading activities, with a reading speed improved to 222 wpm, reflecting a 133.7% increase. In an ocular motility examination, PPC was found to be 19 cm. A reduction in asthenia, insomnia, and RLS were also reported.

The clinical signs and symptoms are summarized in Table 1.

## 3. Discussion

COVID-19 has been frequently associated with a range of ocular symptoms primarily affecting the ocular surface and tear film. These changes may result from the direct action of the virus or from environmental factors related to the disease and its management [18,19]. The most commonly reported symptoms were itchy eyes (8.3%) before and blurred vision (9.2%) after COVID-19 diagnosis [15].

Angiotensin-converting enzyme 2 (ACE2) receptors, the primary entry point for SARS-CoV-2, are expressed in various ocular tissues, with higher concentrations observed in the cornea and limbus, and lower expression levels in the conjunctiva and other ocular structures [20]. Consequently, ocular involvement in COVID-19 has been reported across a range of anatomical sites, including the cornea, lacrimal system, conjunctiva, meibomian glands, sclera and episclera, anterior chamber, retina, and choroid [21,22,23]. Moreover, there is growing evidence linking SARS-CoV-2 infection to corneal small fiber neuropathy. In vivo confocal microscopy (IVCM) studies have identified structural damage to corneal nerve fibers following infection, likely mediated by ACE2 receptor expression in human primary sensory neurons, including trigeminal afferents [24]. For instance, some authors have documented reduced density and morphological alterations of the corneal subbasal nerve plexus post-COVID-19, suggesting a potential mechanism of viral neurotropism via ACE2-mediated entry into corneal innervation pathways [25,26]. These findings support the hypothesis that SARS-CoV-2 may exert direct or immune-mediated effects on corneal sensory nerves, contributing to persistent ocular symptoms in affected individuals [27,28]. Damage to small fibers on the ocular surface alters the afferent branch of the reflex arc that supports tear flow, sharing symptoms and morphological landmarks with dry eye disease (DED) and diabetic neuropathy [29,30]. Furthermore, inflammation of the meibomian glands caused by the virus, the suffering of epithelial cells and the post-COVID-19 increase in tear concentrations of NGF, substance P, and CGRP, common neuromediators involved in ocular surface neuroinflammation, contribute to the development of dry eyes [31,32].

DED is known to be a growing public health problem causing eye discomfort, fatigue, and visual disturbances that interfere with quality of life, including aspects of daily activities and productivity at work [33,34,35,36]. Symptoms worsen especially in the case of prolonged visual activities, such as the use of screens, causing ocular discomfort generally defined as asthenopia or eye strain [37]. In the clinical setting, in addition to symptoms of pain and discomfort of the ocular surface, physicians often detect complaints of blurred vision, even if the patient’s best corrected visual acuity is normal [37]. In this case report, asthenopia was initially attributed to DED and the associated ocular surface inflammation. Although treatment with tear substitutes resulted in improvement in the symptoms and clinical findings of DED, the patient’s visual discomfort during activities such as reading and using electronic devices persisted. This prompted consideration of a broader systemic etiology, particularly in light of the frequent co-occurrence of asthenopia and sleep disturbances, which are well-documented sequelae of SARS-CoV-2 infection [38,39]. Mirza et al. and Bitirgen et al. reported a significant reduction in corneal innervation in post-COVID-19 patients with neurological symptoms, persisting after 3 months from COVID-19 diagnosis, compared to asymptomatic post-COVID-19 patients and healthy controls [26,40]. This may explain why, in addition to some well-documented typical symptoms, such as anosmia and dysgeusia [41], various other neurological manifestations, such as ophthalmoplegia, trigeminal neuropathy, asthenia, restless legs syndrome, and neurophatic corneal pain have been reported in COVID-19 patients [25,29,42,43,44,45,46].

The presence in this patient of exophoria, associated with convergence deficits, must also be considered; asthenopia, or eye strain, is often the result of accommodation and convergence dysfunction, which may be secondary to a neurological deficit that compromises ocular motor control [47]. Post-viral neuroinflammation can alter the neural pathways responsible for ocular alignment and focus, causing fatigue and visual discomfort [48]. Furthermore, intensive use of digital devices during recovery periods can exacerbate these symptoms, especially when associated with underlying neuro-ocular dysregulation.

In this context, reading speed served as an objective functional indicator of visual performance. The marked improvement from 95 to 222 words per minute (a 133.7% increase) following a multimodal treatment regimen suggests that the patient’s visual performance benefited not only from improved neurosensory motor coordination but also cognitive and attentional functions. This observation is consistent with previous studies indicating that both ocular surface dysfunction and neurovisual fatigue can significantly impair reading ability [49,50]. In this case, the use of IReST provided a valuable, patient-centered tool to quantify treatment response and supports the potential of reading speed as a surrogate endpoint in post-COVID neuro-ocular rehabilitation.

RLS is a sensory-motor disorder that causes difficulty in initiating and maintaining sleep: periodic leg movements can contribute to sleep fragmentation in 80% of cases. Dopaminergic dysfunction is currently the most supported and probable pathogenic hypothesis and has a close relationship with metabolic and mood changes also due to the effect of viral infections [44]. For this reason, the onset of RLS affects sleep efficiency and quality of life, causing lack of energy during the day, mood swings, and difficulty concentrating and performing work and daily activities [46]. In view of these manifestations, it is assumed that functional and behavioral changes caused by COVID-19 worsen or trigger RLS symptoms in predisposed people [51]. In some cases, RLS has been reported as a direct consequence of SARS-CoV-2 infection, but it has never been associated with asthenopia, which is also partly related to autonomic dysfunction and neurological disorders [51,52]. Indeed, it has been shown that asthenopia, or eye strain, can be associated with dopaminergic disorders such as Parkinson’s disease (PD). Dopamine plays a crucial role in several visual functions, including oculomotor control, contrast sensitivity, and light adaptation. Therefore, its deficiency, particularly in the retina and visual cortex, can lead to visual disturbances and asthenopia [53,54].

The persistence of symptoms such as DED, visual acuity, insomnia, and RLS in this patient suggests a common multifactorial etiology rooted in both direct viral effects and post-viral neurological and immune dysregulation.

The pathological mechanisms underlying these symptoms can be traced back to three fundamental areas:

1. Neuroinflammation: SARS-CoV-2 virus can directly affect the central nervous system, leading to neuroinflammation. Several studies have shown that SARS-CoV-2 may cross the Blood–Brain Barrier (BBB) via the ACE2 receptor, leading to the activation of glial cells and the release of pro-inflammatory cytokines [55]. This process may explain many of the neuropsychiatric symptoms seen in LC, including fatigue, sleep disturbances, and RLS. Furthermore, the inflammatory response may contribute to ocular symptoms, as immune dysregulation can affect the ocular surface, tear film, and neuromotor balance, causing dry mouth and visual asthenopia.
2. Autonomic Nervous System Dysfunction: There is growing evidence suggesting that LC may involve dysautonomia, a condition characterized by the dysfunction of the autonomic nervous system [56]. Dysautonomia can lead to altered tear production and increased ocular dryness, as well as disturbances in sleep patterns, which could explain the patient’s insomnia and RLS. Furthermore, autonomic dysfunction could exacerbate fatigue and asthenopia, as the body’s response to stress and physical activity is impaired.
3. Immune Dysregulation and Post-Viral Fatigue: Post-viral fatigue is a hallmark of LC and may be mediated by ongoing immune activation. This includes increased levels of cytokines such as interleukins (IL-6, IL-1β), which have been linked to fatigue, mood disturbances, and cognitive dysfunction in LC patients [57]. Immune dysregulation could contribute to the persistence of symptoms like DED and visual asthenopia, as the inflammation in peripheral tissues, including the ocular surface, may be sustained even after the initial infection resolves.

In this case report, we obtained a resolution of the clinical symptoms with medical devices without using drugs with potential unwanted effects. The morning administration of citicoline associated with other components and the evening administration of a melatonin-based supplement for six weeks, in addition to the prolonged use of multi-action tear substitutes, allowed for the resolution of general and visual asthenia, but also of sleep disturbances and RLS.

Given the positive response to this combination therapy (tear substitutes, choline, vitamins, melatonin), it seems that these systemic treatments can address not only ocular symptoms but also the broader manifestations of LC.

The lacrimal glands are innervated by sympathetic nerves and parasympathetic nerves, which predominantly regulate their secretions [58]. The parasympathetic nervous system releases ACh, which acts on muscarinic and nicotinic receptors. Muscarinic receptors are important mediators of lacrimal and salivary gland secretions [59], and their presence has also been reported in the cornea, conjunctiva, and meibomian glands [60,61]. Choline is a precursor of ACh, so its deficiency may contribute to the development of dry eye syndrome by reducing parasympathetic tone [33]. Another key pathological mechanism underlying dry eye syndrome is inflammation, as demonstrated by T-cell infiltration and elevated expression of immune markers such as CD3, CD4, and CD8, along with activation markers including CD11a and HLA-DR. This inflammatory milieu is further characterized by increased levels of pro-inflammatory cytokines such as interleukin IL-1, IL-6, IL-8, and tumor necrosis factor-alpha (TNF-α) on the ocular surface [62]. Choline deficiency has been reported to cause oxidative damage in various organs [63], increase inflammation in dry eye syndrome, and delay wound healing on the ocular surface. Therefore, choline deficiency may increase inflammation in dry eye syndrome and delay the healing of ocular surface lesions [64]. Choline supplementation suppresses tissue inflammation and oxidative damage [65] and has been suggested as a novel therapeutic method for the control of immune inflammation [66]. Acetylcholine (ACh)-stimulated nicotinic receptors on inflammatory cells have been shown to inhibit the release of pro-inflammatory cytokines in a concentration-dependent manner [67]. Choline plays a multifaceted role in cellular metabolism and protection, contributing to tissue repair after injury and mitigating hypoxia-induced damage to blood vessels and vascular endothelial cells [64]. It also exhibits analgesic properties through the activation of α7 nicotinic acetylcholine receptors [68]. Moreover, choline serves as a precursor to phosphatidylcholine, a major structural component of cell membranes and the tear film [64]. Choline alfoscerate, a choline-containing surfactant, enhances tear film stability by supporting both the aqueous and lipid layers [56].

We also believe that the vitamin supplements, in particular B vitamins (B1, B5, B6, B12), anthocyanosides, choline, reishi, CoQ10, and vitamins C, D3, and E, contained in the Colinplus Delta^®^ product plays a fundamental role in addressing systemic symptoms, including RLS, asthenia, and asthenopia [69,70,71,72]. In fact, the known characteristics of its components can have useful effects in post-COVID recovery.

Vitamin B complex (B1, B5, B6, B12) plays a crucial role in nerve health, energy production, and the functioning of the nervous system. They could contribute to reducing fatigue and supporting general recovery from neurological symptoms, possibly improving RLS and general wellbeing [73].

Anthocyanosides are antioxidants derived from fruits and plants, especially berries, and they help in protecting the blood vessels and supporting circulation. This can help manage symptoms related to asthenopia and general fatigue [74,75]; high concentration of choline, known for its role in maintaining the integrity of cell membranes and supporting brain function, could help improve cognitive functions and reduce fatigue and feelings of heaviness in the limbs, which are common in RLS [76].

Reishi (Ganoderma lucidum) is a mushroom used in traditional medicine for its potential immune-modulating and anti-fatigue effects. It may help manage sleep disorders, fatigue, and even improve general wellbeing in post-viral recovery [77].

Vitamin C, D3, and E support immune function, help in tissue repair, and have antioxidant properties that may support the recovery of ocular health and overall energy levels, potentially easing both ocular and systemic symptoms like fatigue and sleep disturbances [78].

CoQ10 may help lower low-density lipoprotein cholesterol and cumulative cholesterol levels in those with diabetes, reducing their chance of heart disease, Parkinson’s disease, and statin-induced myopathy [79]. CoQ10 was shown in one test to prevent migraines compared to a placebo [79]. In addition, CoQ10 offers a promising potential treatment for improving sleep quality by supporting cellular energy production, reducing oxidative stress, and enhancing mitochondrial function [80].

The generic antioxidant effects of SODs are beneficial under all tested conditions, from ocular and cardiovascular diseases to neurodegenerative disorders and metabolic diseases, including diabetes and its complications and obesity [81,82]. Pyrroloquinoline quinone (PQQ) showed significant improvements in sleepiness at awakening, sleep onset and maintenance, and sleep duration [83].

Zinc is an essential trace element involved in the regulation of obesity-related hormones, including insulin, leptin, and thyroid hormones, and it contributes to numerous metabolic pathways [84,85]. It also functions as a cofactor in the synthesis and metabolism of key neurotransmitters such as glutamate and gamma-aminobutyric acid (GABA) which are critical for sleep regulation [86]. Additionally, zinc supports the production of melatonin, a hormone central to the circadian rhythm and sleep–wake cycle [87]. Its role in maintaining immune competence is well established, and zinc deficiency has been associated with increased susceptibility to infections and inflammation, both of which may contribute to sleep disturbances [86]. Moreover, zinc exhibits antioxidant properties, helping to counteract oxidative stress [88].

Selenium is an essential trace element naturally present in many foods and a vital component of 25 known selenoproteins, including thioredoxin reductases, glutathione peroxidases, and selenoprotein P. These selenoproteins are involved in key biological functions such as thyroid hormone metabolism, DNA synthesis, reproductive health, and protection against oxidative stress and infection [89]. Low serum selenium levels have been associated with the onset and severity of Restless Legs Syndrome (RLS), with clinical studies demonstrating symptom improvement following selenium supplementation [90]. Furthermore, selenium may help alleviate asthenopia and systemic fatigue by supporting mitochondrial function and reducing oxidative stress—mechanisms particularly relevant in the context of post-viral or inflammatory fatigue syndromes, including long COVID [89,91].

Melatonin was used in the treatment plan and plays a pivotal role in treating sleep disorders and can potentially alleviate symptoms of insomnia and help regulate circadian rhythms. In long COVID cases, where sleep disturbances are common, melatonin may help restore restful sleep, which could have a secondary positive effect on RLS [92].

The near-simultaneous presentation of RLS, dry eye, asthenopia, fatigue, and sleep disturbances, as well as the positive outcome of all clinical manifestations after six weeks of treatment, suggests a clear association with the recent history of COVID-19 infection and a correlation between the various symptoms.

The originality of the case consists of the possible association of asthenopia and RLS with other symptoms more commonly reported following SARS-CoV-2 infection, such as sleep disorders and eye burning.

The therapeutic results obtained in restless legs syndrome (RLS) with the oral supplementation of choline and other micronutrients may suggest supplementation with these products can treat mild to moderate forms of ocular and systemic asthenia and sleep disturbances, even in cases unrelated to SARS-CoV-2 infection. However, the lack of electrophysiological and neuroimaging assessments limits the objective characterization of patients’ neurological manifestations and prevents the exclusion of alternative or coexisting etiologies. To strengthen the clinical relevance of these findings, future studies with larger patient cohorts and controlled methodologies are needed to confirm the observed associations and evaluate the efficacy of the proposed therapeutic strategy on individual pathological manifestations.

## 4. Conclusions

The presented clinical case outlines a compelling connection between various symptoms following recovery from COVID-19, including dry eye (DED), general fatigue and asthenopia, sleep disturbances, and RLS. The persistence of these clinical manifestations after COVID-19 infection may be attributed to complex etiopathogenetic mechanisms involving neuroinflammation, vascular dysregulation, and autonomic dysfunction. The key aspect of this case is the hypothesis that these symptoms may be interconnected and manageable with a therapeutic approach involving a combination of tear substitutes, choline, vitamins, and melatonin. Therapeutic strategies that combine nutritional support for ocular surface health with neuroprotective agents such as choline, melatonin, and vitamin complexes may promote symptom resolution by targeting both peripheral and central nervous system alterations. Further studies on other similar cases are needed to confirm the cause–effect relationship between the observed disorders and COVID-19, the association of the different pathological alterations in response to unique pathogenic mechanisms, and the validity of the proposed therapeutic approach.

## Figures and Tables

**Table 1:** Clinical case: signs and symptoms of ocular surface disease, asthenopia, and neurological manifestations at time 0, 10 days, and 42 days.

	Fluotest (NEI Score)		TBUT		Schirmer Test I (mm)		OSDI Score	Reading Speed (wpm)	Proximal Point of Convergence (PPC) (cm)	Neurological Disorders
	Right	Left	Right	Left	Right	Left				
Time 0	2	3	3”	2”	8	6	36	95	38	Sleep Disorders, Asthenia, and Restless Legs Syndrome
10 Days	0	0	6”	5”	10	9	25	128	36	Sleep Disorders, Asthenia, and Restless Legs Syndrome
42 Days	0	0	9”	8”	10	9	11	222	19	None

## Data Availability

All data are provided in the main text.
